# Long-term outcome of PDT for local failure after CRT or RT for oesophageal cancer

**DOI:** 10.1007/s00535-025-02316-x

**Published:** 2025-11-12

**Authors:** Takahiro Horimatsu, Tomonori Yano, Yoshinobu Yamamoto, Hiromi Kataoka, Yohei Yabuuchi, Ryu Ishihara, Hiroi Kasai, Ryuji Uozumi, Harue Tada, Manabu Muto

**Affiliations:** 1 Department of Medical Oncology, Graduate School of Medicine, Kyoto University, Kyoto, Japan; 2 Institute for Advancement of Clinical and Translational Science, Kyoto University Hospital, Kyoto, Japan; 3 Department of Gastroenterology and Endoscopy, National Cancer Centre Hospital East, Kashiwa, Japan; 4 Department of Gastroenterological Oncology, Hyogo Cancer Centre, Hyogo, Japan; 5 Department of Gastroenterology and Metabolism, Nagoya City University Graduate School of Medical Sciences, Nagoya, Japan; 6 Division of Endoscopy, Shizuoka Cancer Centre, Shizuoka, Japan; 7 Department of Gastrointestinal Oncology, Osaka International Cancer Institute, Osaka, Japan

**Keywords:** Esophageal cancer, Photodynamic therapy, Long-term outcome

## Abstract

**Background:**

The long-term local control and effect of photodynamic therapy (PDT) using talaporfin sodium and a diode laser (talaporfin PDT) on overall survival (OS) for local failure after chemoradiotherapy (CRT) for esophageal cancer are unknown. Here, we present a 5-year survival analysis for talaporfin PDT.

**Methods:**

This was a prospective follow-up analysis of an open-label, multicenter, phase 2 study of local failure after CRT or radiotherapy in patients who received talaporfin PDT. The primary endpoint was the overall OS. The secondary endpoints were progression-free survival (PFS), local progression-free survival (L-PFS) with local progression or recurrence and death as events, and local time to progression (L-TTP) with only local progression or recurrence as events.

**Results:**

Between November 2012 and December 2013, 26 patients with oesophageal squamous cell carcinoma underwent talaporfin PDT. The baseline T stages were cT1 in 14 patients, cT2 in 6 patients, and cT3 in 6 patients. Pre-PDT T stages were cT1b for 19 and cT2 for 7 patients, and no lymph nodes or distant metastases were detected. At a median 6.8-year follow-up, the median OS and 5-year OS rates were 4.2 years (95% confidence interval [CI]: 1.6–7.3) and 40.6% (95% CI: 21.7–58.7), respectively. The median PFS and L-PFS were 1.1 and 2.1 years, respectively. The 5-year local progression-free rate was 84.9%. No treatment-related deaths occurred.

**Conclusion:**

Talaporfin PDT for patients with oesophageal cancer with local failure after CRT can achieve long-term local complete response and long-term survival as a minimally invasive salvage treatment.

**Trial registration number:**

UMIN000009184.

## Introduction

Oesophageal cancer is the 11th leading cause of cancer-related deaths worldwide in 2022 (3.3%) [1] and is one of the most intractable cancers. The prognosis is reported to be extremely poor, with 5-year overall survival rates of 20% and 15% in the United States and United Kingdom, respectively [2]. According to the National Comprehensive Cancer Network guidelines, preoperative chemoradiotherapy (CRT) followed by surgery or definitive CRT is the standard of care for advanced oesophageal squamous cell carcinoma [3]. Compared with surgery, CRT has several merits that can preserve the organ and its function. However, CRT has the disadvantage of a high local residue or recurrence rate (30–40%), although it shows a high response rate [4]. Furthermore, residual or recurrent tumours usually grow rapidly; thus, the timing of salvage treatment is important for selecting modalities and their outcomes.

Surgery is usually indicated as a potentially curative salvage treatment for locoregional failure after definitive CRT; however, morbidity and mortality are high [5]. Although the 5-year survival rate of salvage surgery after CRT has been reported to be 25–38% [6–15], hospital deaths due to postoperative complications were high (3.7–22%) [8–11]. Chemotherapy such as taxane or immune checkpoint inhibitors for failure after CRT is not curative but palliative, because the complete response (CR) rate in this setting was reported to be 0–6% [12–19]. Therefore, there is an urgent need to develop less invasive and curative salvage treatment for local residue or recurrence after CRT.

Therefore, we developed salvage photodynamic therapy (PDT) as a less invasive curative treatment. We previously reported that PDT with talaporfin sodium and a diode laser (talaporfin PDT) could achieve a high local CR (L-CR) in patients with local residue or recurrent oesophageal cancer after CRT or radiotherapy (RT), leading to Pharmaceutical and Medical Devices Agency (PMDA) approval in Japan in 2015 [20]. Herein, we reported the long-term local complete control rate of talaporfin PDT and its effect on overall survival (OS) at the 5-year follow-up.

## Methods

### Ethics statements

The study protocol and all amendments for both the phase 2 trial and the prospective follow-up study were approved by the institutional review boards and ethics committees of all participating institutions. The phase 2 study was conducted in accordance with Good Clinical Practice guidelines, and the follow-up study was conducted in accordance with the ethical principles of the Declaration of Helsinki. Written informed consent was obtained from all patients prior to enrolment in the phase 2 trial and follow-up study. This trial was registered in the UMIN Clinical Trials Registry (UMIN000009184).

### Study design and participants

This was a prospective follow-up study of an open-label, multicentre, phase 2 trial conducted at seven sites in Japan [20]. Patients aged ≥ 20 years with local failure after CRT or RT (≥ 50 Gy) for oesophageal cancer were included in the phase 2 trial. The eligibility criteria and exclusion criteria for the phase 2 trial have been described previously [20].

### Procedures

**Fig. 1:**
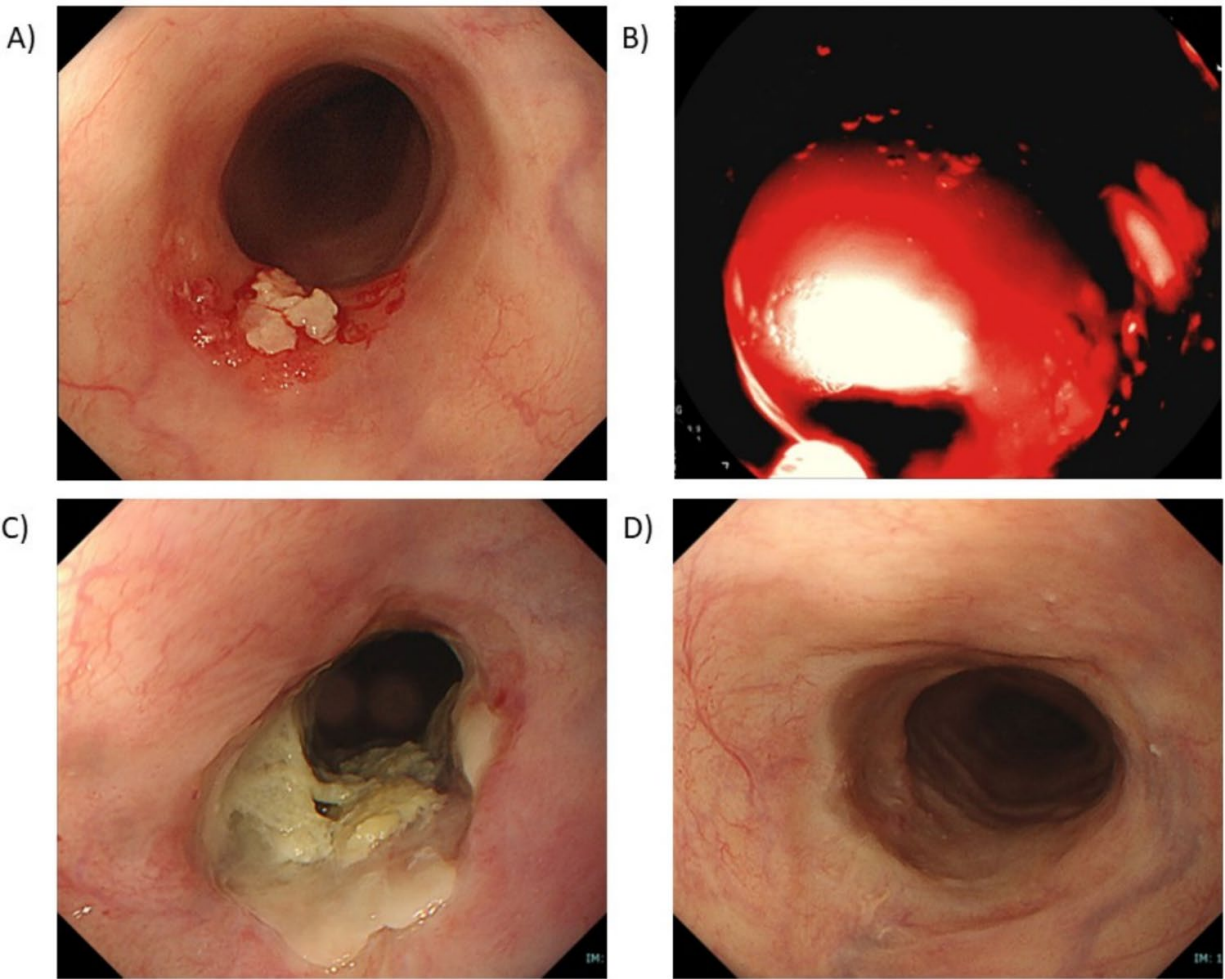
Patient who achieved complete response with photodynamic therapy. **A** Local residue was detected after chemoradiotherapy. **B** Laser irradiation during PD. **C** Seven days after PDT, ischaemic change was observed at the laser-irradiated site. **D** CR was achieved 10 weeks after PDT

Talaporfin PDT (dose of talaporfin sodium, 40 mg/m^2^) was administered intravenously followed by diode laser irradiation at a 664-nm wavelength 4–6 h after administration (Fig. 1). The fluence of the diode laser was set at 100 J/cm^2^ at a fluence rate of 150 mW/cm^2^. The diode laser is delivered via a frontal light distributor through the operative channel of the endoscope. The plastic attachment was fitted in front of the scope to maintain the distance between the tip of the scope and lesion. If the lesions were larger than 1 cm^2^, multiple irradiated areas were overlapped to cover the entire lesion. Endoscopic observation was mandatory on the following day, and if an obvious residual tumour was found, additional laser irradiation was indicated. ‘Obvious residual tumour’ refers to (1) presence of a residual to incomplete submucosal tumour-like protruded component, (2) presence of a neoplastic mucosa or ulcer, and (3) absence of oedematous mucosa with redness or dark blue discoloration due irradiation.

The endoscopic examination was repeated at least once every 2 weeks until the response was confirmed. Endoscopic evaluation of the primary site of gastrointestinal cancer during chemotherapy and/or radiotherapy has not been adopted by the Response Evaluation Criteria in Solid Tumours because it is not objective. Clinical T stage before PDT was evaluated by endoscopy, endoscopic ultrasound (EUS), and computed tomography (CT) of the chest. However, evaluation of the primary site of gastrointestinal cancer using CT is difficult, especially for complete response. To clinically visualise the complete response of the primary site of gastrointestinal cancer, direct observation using endoscopy is reasonable. The criteria for a local complete response in this study were as follows: (1) no residual tumour observed, (2) disappearance of the post-PDT ulcer and confirmed scar formation, and (3) histological disappearance of cancer cells. When L-CR was confirmed (cL-CR) or local progressive disease (L-PD) was confirmed, endoscopic surveillance was performed every 4 weeks until 24 weeks after PDT. After 24 weeks, endoscopic surveillance was performed every 3 months for 2 years and every 6 months thereafter until a 5-year follow-up to determine local progression and recurrence. An endoscopic biopsy was performed when progression was suspected to histologically confirm local recurrence.

Local efficacy was classified by endoscopic evaluation as L-CR, L-PD, local nonCR/nonPD (L-nonCR/nonPD), or not evaluable (NE) at each evaluation. The criteria for L-CR were as follows: (1) no residual tumour observed; (2) disappearance of the post-PDT ulcer and confirmed scar formation; (3) histological disappearance of cancer cells. cL-CR was defined as a continuation of L-CR status for 4 weeks or longer. Conversely, the criteria for L-PD were as follows: (1) definitive progression compared with the lesion before enrolment or (2) recurrence after achieving L-CR with PDT. When neither the L-CR nor L-PD criteria were met, the lesions were classified as L-nonCR/nonPD. Furthermore, when endoscopic examination and tissue diagnosis by biopsy could not be performed, or when the lesion could not be classified as L-CR, L-PD, or L-nonCR/nonPD, the lesions were classified as NE.

Contrast-enhanced computed tomography with a 5 mm slice thickness of the neck, chest, and abdomen was performed every 6 months for 2 years and then at 2, 2.5, 3, and 5 years to determine the progression and recurrence of lymph nodes and distant organs. Lymph node metastasis was clinically diagnosed if the lymph node was visible and > 10 mm in short diameter and PET scan was performed as needed, and a decision was made based on comprehensive evaluation. Distant metastasis was defined as detection of a new space-occupying lesion.

Patients were assessed by physical examinations and measurements of haematological and biochemical variables in the blood every 3 months until 2 years and then at 2, 2.5, 3, and 5 years after PDT. Adverse events and toxicity were evaluated in all treated patients using the Common Terminology Criteria for Adverse Events, version 4.0 [21].

### Outcomes

The primary outcome of this follow-up study was OS, which was measured from the date of enrolment in the original study until death from any cause. If no event was observed, the period was censored on the last day on which the patient’s survival was confirmed. The secondary outcomes were local progression-free survival (L-PFS), progression-free survival (PFS), and local time to progression (L-TTP). L-PFS was measured from the date of enrolment to the first date of L-PD confirmation or death from any cause. If no events were observed, the period was censored on the last endoscopic evaluation date. PFS was measured from the date of enrolment to the first date of confirmation of L-PD, new metastasis, progression to a lymph node or distal organ, or death from any cause. L-TTP was measured from the date of enrolment to the first date of L-PD confirmation. If no events were observed, the period was censored on the date of the last endoscopic evaluation.

### Statistical analysis

The original study design for the phase 2 trial has been described previously [20]. In this study, OS, L-PFS, PFS, and L-TTP rates were estimated from the Kaplan–Meier method with 95% confidence intervals (CIs) using complementary log–log transformation and Greenwood’s variance. Median estimates with 95% CIs were computed using the Brookmeyer and Crowley method. The follow-up period was calculated using reverse Kaplan–Meier estimates [22]. Analyses were performed using SAS software (version 9.4; SAS Institute, Cary, NC).

## Results

### Patient characteristics

All 26 patients treated with talaporfin PDT in the phase 2 study were analysed, and their long-term outcomes were examined. Patient characteristics before PDT and CRT are shown in Table 1. Solitary failure lesions were found in 24 of 26 patients, while the other 2 patients had 2 lesions each. All patients (26 men; median age, 71.5 years) were histologically diagnosed with squamous cell carcinoma.

**Table 1:** Baseline patient characteristics before PDT

Characteristic	Total (*N* = 26)
Median age	71.5
(range)	51–86
Sex	
Male	26
Female	0
Histology finding	
Squamous cell carcinoma	26
Adenocarcinoma	0
Base line T stage	
T1	14
T2	6
T3	6
Base line N stage	
N0	18
N1	3
N2	5
Pre-PDT T stage	
T1a	0
T1b	19
T2	7
Pre-PDT N stage	
N0	26
N1/N2/N3	0
Pre-PDT M stage	
M0	26
M1	0
Pre-PDT TNM stage	
T1bN0M0	19
T2N0M0	7
Best response to talaporfin PDT	
L-CR	23
L-nonCR/nonPD	3
L-PD	0
ECOG-PS score	
0	19
1	6
2	1
3	0
4	0

PDT, photodynamic therapy; TNM, tumour, node, metastasis; ECOG-PS, Eastern Cooperative Oncology Group-performance status; L-CR, local complete response; L-nonCR/nonPD, local non-complete response/non-progression disease; L-PD, local progression disease

Before CRT Base line Stage, T1 was in 14, T2 was in 6, and T3 was in 6 lesions, respectively, and N0 was in 18, N1 was in 3, and N2 was in 5 lesions, respectively. Pre-PDT stage, 19 patients had T1 stage oesophageal cancer, and 7 patients had T2 oesophageal cancer. No lymph node or distant metastasis was observed in any case. The best responses to talaporfin PDT were CR in 23 patients and L-nonCR/nonPD in three patients. None of the patients showed L-PD as the best response to talaporfin PDT.

### Follow-up and outcomes

**Fig. 2:**
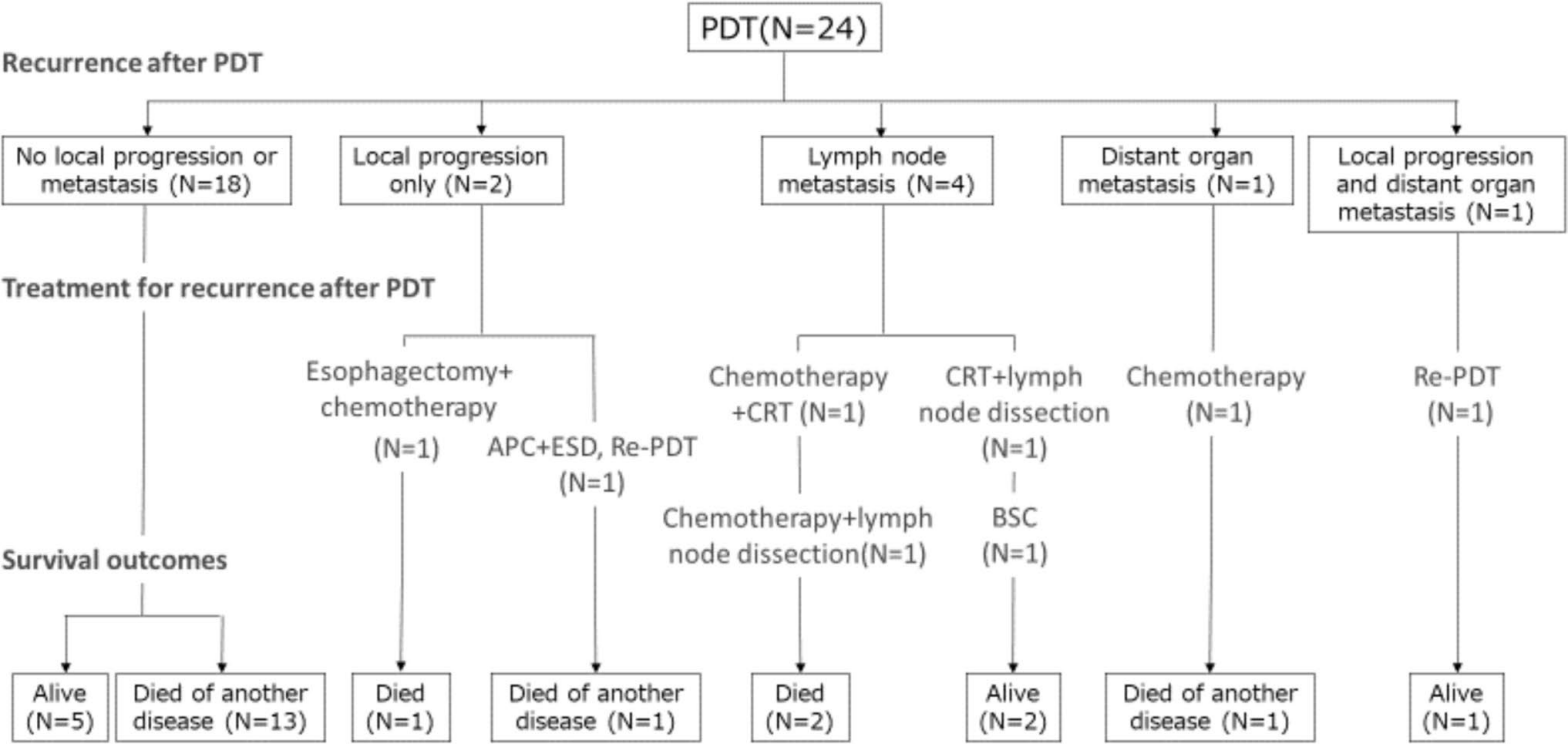
Patients’ clinical course after PDT. PDT, photodynamic therapy; APC, argon plasma coagulation; ESD, endoscopic submucosal dissection; EMR, endoscopic mucosal resection; CRT, chemoradiotherapy; BSC, best supportive care

The median follow-up period was 6.8 years (95% CI, 6.5–7.5). The posttreatment outcomes after PDT are presented in Fig. 2. During the follow-up period, 18 patients (69.2%) did not show local progression or metastasis. Among them, 5 patients were alive, and 13 died of another disease. The details were as follows: lung cancer 2, pulmonary embolism 1, pneumonia 4, bladder cancer 2, unknown 2, and death from other cancers, 1. Two patients experienced local disease progression. Although these patients could be treated endoscopically, they died of another disease. Four patients developed lymph node metastasis. Three patients were treated with chemotherapy, CRT, and/or lymph node dissection. Among them, 2 patients died of oesophageal cancer and 2 patients were still alive. One patient developed distant metastasis, was treated with palliative chemotherapy, and died from another disease. One patient developed local progression and distant metastases and was treated with again with PDT. Finally, only 3 patients died of oesophageal cancer.

Three of the 26 patients (11.5%) required additional treatment for the local progression of the primary lesions. Endoscopic treatment was indicated in 2 patients, and oesophagectomy was indicated in 1 patient. Four patients required additional treatment for lymph node or distant metastases. Among the 8 cases with lymph node metastasis at baseline, only one case developed lymph node metastasis after PDT during a median follow-up of 4.1 years. Furthermore, this case involved recurrence of lymph node metastasis outside the irradiation field, not in the lymph nodes present at baseline.

**Fig. 3:**
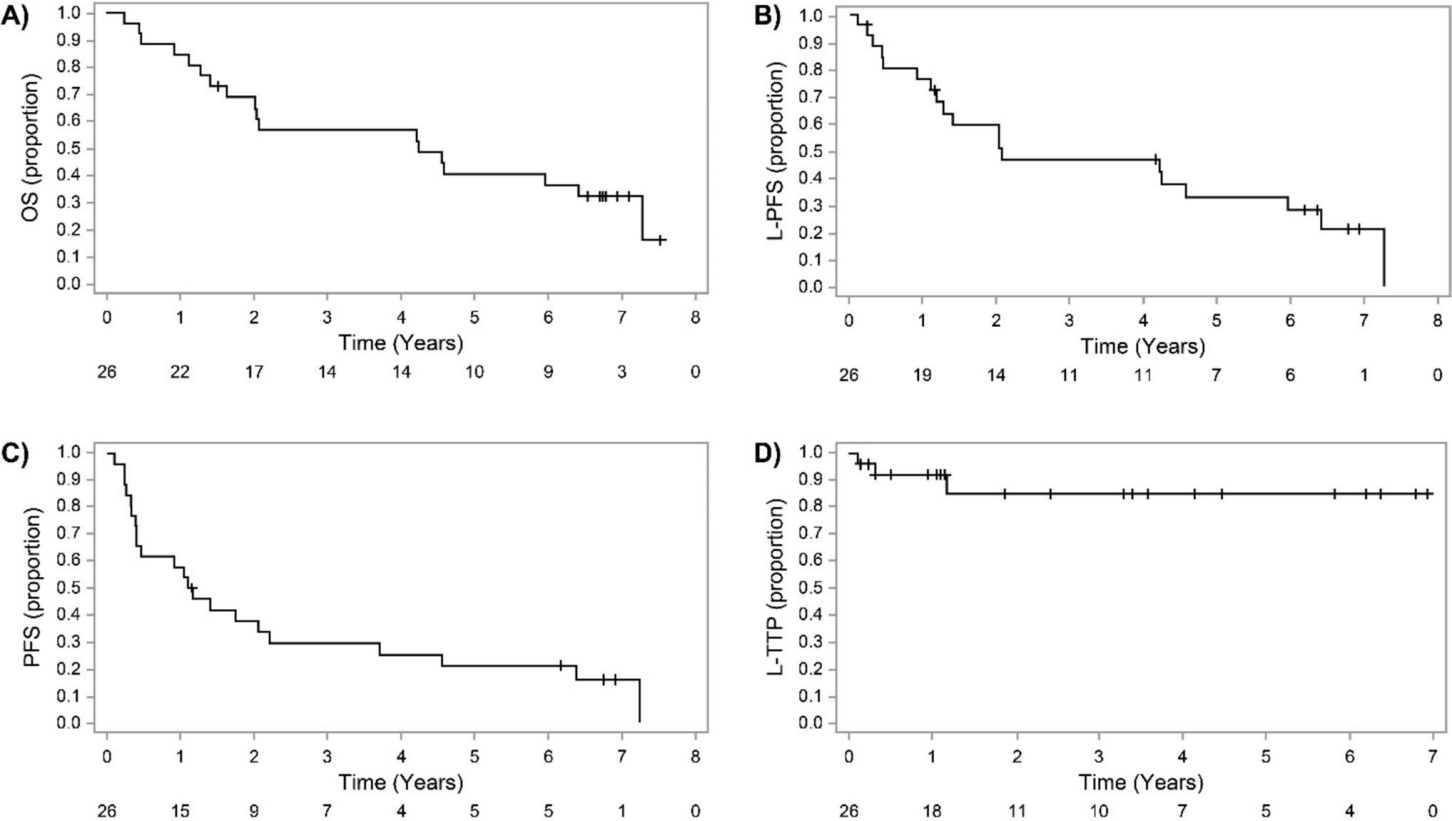
Survival curves in this study. **A** Overall survival (OS), **B** local progression-free survival (L-PFS), **C** progression-free survival (PFS), **D** local time to progression (L-TTP)

Survival curves are shown in Fig. 3. The median OS of the 26 patients was 4.2 years (95% CI: 1.6–7.3), and the 5-year OS rate was 40.6% (95% CI: 21.7–58.7) (Fig. 3A). At the time of this analysis, 18 of the 26 patients (61.5%) had died. However, only 3 of the 18 patients died of oesophageal cancer, while the others died of other diseases. Nineteen events were confirmed in the L-PFS curve, and the median L-PFS was 2.1 years (95% CI: 1.2–6.0) (Fig. 3B). Twenty-two events were confirmed for the PFS curve; the median PFS was 1.1 years (95% CI: 0.4–2.2), and the 5-year PFS rate was 20.8% (95% CI: 7.7–38.3) (Fig. 3C). Regarding L-TTP, only 3 events occurred; thus, the median L-TTP was undefined for this follow-up period (Fig. 3D). The 5-year L-TTP rate was 84.9% (95% CI: 58.3–95.2).

In the analyses by reason of PDT, the 5-year OS for residual lesions was 20% (95% CI: 0.8–58.2) and for recurrent lesions is 45.7% (95% CI: 23.7–65.4), which is not a significant difference (*P*-value: 0.382).

## Discussion

There have been no prospective reports investigating long-term local control by PDT and its effect on long-term survival after CRT failure for oesophageal cancer. Usually, salvage surgical treatment is indicated in such cases; however, the morbidity and mortality rates associated with salvage surgery are high. In contrast, PDT is a non-surgical, less invasive treatment that shows a high local complete response (70–90%) for local failure after chemoradiotherapy or radiotherapy for oesophageal cancer. In other words, a complete rather than partial response is the most useful benefit of PDT However, some experts were sceptical that photodynamic therapy may not be effective in terms of long-term prognosis because it is a local treatment. This study aimed to address clinical questions regarding local control (complete response) and long-term outcomes in patients with oesophageal cancer treated with photodynamic therapy as salvage treatment after chemoradiotherapy.

Since PDT is a local treatment, it is unclear how much it contributes to the OS of patients with local failure after CRT or RT for oesophageal cancer. Oesophageal cancer is one of the most intractable cancers and has a poor prognosis. Even patients with T1b oesophageal cancer are at high risk of lymph node metastasis (20–50%). The prognosis of patients who failed CRT or RT with local treatment alone was considered poor.

In this prospective 5-year follow-up analysis, talaporfin PDT showed excellent local complete control because only 3 cases of local progression (11.5%) occurred in 26 patients. The 5-year L-TTP rate after talaporfin PDT was 84.9%. This result indicated that talaporfin PDT resulted in effective long-term local complete control. Furthermore, 76.9% (20/26) of the patients did not develop lymph node or distant metastasis, resulting in a median OS of 4.2 years and a 5-year OS rate of 40.6%. Previous research indicated that talaporfin photodynamic therapy (PDT) has a favourable safety profile [20]. To the best of our knowledge, this is the first prospective study to clarify the long-term local complete control rate of salvage talaporfin PDT and its effect on long-term prognosis.

We previously reported that achieving L-CR by salvage PDT was associated with a better prognosis than that of nonCR cases [23]. Minamide et al. also reported that L-CR induced by PDT is associated with longer survival [24]. These reports indicate that local complete control is important to improve patient survival after PDT. However, these reports were made by a single institute and were retrospective. In this multicentre prospective study, we showed not only a high L-CR rate with talaporfin PDT, but also a long CR effect. These results indicate that longer local complete control might be associated with longer OS.

Local recurrence may occur even in patients who can achieve L-CR. In the case of radiotherapy, radical irradiation was performed, and re-irradiation was not possible because of organ toxicity. In contrast, PDT can be re-irradiated. We recently reported that the L-CR rate after repeated PDT was 56.3% and that repeated PDT did not result in any severe adverse events [25]. Yamashita et al. also reported that the L-CR rate after the second PDT session was 40.7% and that there were no severe adverse events [26]. These results indicate that repeated PDT can provide high L-CR even in situations where salvage treatment options are limited, and it is clinically significant as an organ- and function-preserving treatment.

Patients with local, recurrent, or persistent oesophageal cancer after curative definitive CRT are potential candidates for salvage surgery. However, salvage surgery is associated with a high risk of morbidity, including pulmonary complications (29.3%), anastomotic leaks (17.2%), and cardiovascular complications (6.7%) [4]. The 90-day mortality rate was 8.8%. Therefore, minimally invasive and highly curative salvage treatment is required.

In this study, 8 patients (30.8%) had lymph node metastases at baseline, but before PDT, none had lymph node metastasis. Therefore, it is possible that lymph node metastasis was already controlled with CRT or RT. Among the 8 cases, only 1 case showed lymph node metastasis after PDT, but the lymph node metastasis was not present at baseline and was an extraradiation field.

In the T stage before PDT, T1b accounted for 73.1% (19/26) of the cases. Therefore, it is possible that the prognosis was better after PDT. In contrast, a meta-analysis reported that the 5-year OS of salvage surgery after definitive CRT was 24.1% [4]. Sudo et al. reported that among patients with local relapse by definitive CRT, the median OS and 5-year OS rate after salvage surgery were 58.6 months and 45% (95% CI, 26–79%), respectively [27]. Furthermore, Swisher et al. reported that early pathological stage (T1N0, T2 N0) was associated with improved prognosis after salvage surgery [6]. This study showed that the prognosis of talaporfin PDT was comparable to that of salvage surgery, even considering the possibility of controlled lymph node metastasis and the high rate of T1b cases.

Importantly, talaporfin PDT can preserve the oesophagus. No treatment-related adverse events were associated with talaporfin PDT. Therefore, talaporfin PDT may be the ultimate minimally invasive treatment. Considering the loss of organs and high morbidity and mortality rates in salvage surgery, we believe that talaporfin PDT is an important option for salvage treatment. To perform talaporfin PDT, quality control of the procedure is important to achieve L-CR. First, laser irradiation must be precisely applied to the oesophagus, where oesophageal peristalsis is present. Second, if irradiation is judged to be insufficient on the next day’s observation, the endoscopist should perform laser irradiation again to achieve an L-CR. For these reasons, at the time of PMDA approval in Japan, it became a condition of insurance coverage in which a training course was conducted, and the endoscopists were certified.

This study had several limitations. First, this was a single-arm study and was not compared with those of other salvage treatments. Other salvage treatments after failure of definitive CRT for oesophageal cancer include salvage surgery and chemotherapy such as taxane or immune checkpoint inhibitors. However, it might be difficult to conduct a randomised controlled trial with salvage surgery and PDT because their invasiveness is significantly different. In addition, comparative studies with other endoscopic resections (ERs) are also difficult because ER for local failure of T1b and T2 is difficult to perform, and there is no prospective data on ER in a salvage setting. Second, PDT was performed by certified and skilled endoscopists who are experts in PDT treatment. To achieve high CR, laser irradiation must be precisely targeted to the lesion, as previously mentioned. Manuals and training sessions for PDT procedures were implemented to control the PDT quality. This educational and training system is extremely important for widespread clinical use of salvage PDT. Third, this study included only a small number of patients. However, even in a limited number of cases, a high CR ratio represents a significant benefit for patients.

Furthermore, recent in vivo studies in mice have demonstrated that combined treatment with ICI and talaporfin PDT enhances the antitumor effect. Future research should consider the combination treatment with ICI in addition to PDT [28].

In conclusion, talaporfin PDT for local failure after definitive CRT or RT for oesophageal cancer can achieve a long-term local CR and long-term survival. It is an effective and minimally invasive salvage treatment that preserves organs.
